# Loop-Mediated Isothermal Amplification-Integrated CRISPR Methods for Infectious Disease Diagnosis at Point of Care

**DOI:** 10.1021/acsomega.3c04422

**Published:** 2023-11-07

**Authors:** Defne Yigci, Nazente Atçeken, Ali K. Yetisen, Savas Tasoglu

**Affiliations:** † School of Medicine, Koç University, Istanbul 34450, Turkey; ‡ Koç University Translational Medicine Research Center (KUTTAM), Koç University, Istanbul 34450, Turkey; § Department of Chemical Engineering, Imperial College London, London SW7 2AZ, U.K.; ∥ Boğaziçi Institute of Biomedical Engineering, Boğaziçi University, Istanbul 34684, Turkey; 5 Koç University Arçelik Research Center for Creative Industries (KUAR), Koç University, Istanbul 34450, Turkey; 6 Physical Intelligence Department, Max Planck Institute for Intelligent Systems, Stuttgart 70569, Germany

## Abstract

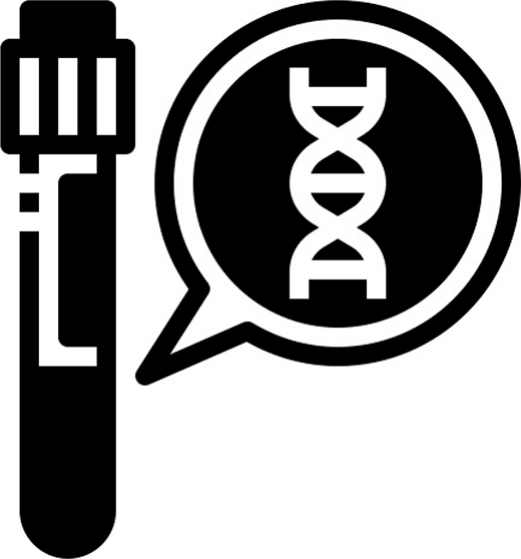

Infectious diseases continue to pose an imminent threat to global public health, leading to high numbers of deaths every year and disproportionately impacting developing countries where access to healthcare is limited. Biological, environmental, and social phenomena, including climate change, globalization, increased population density, and social inequity, contribute to the emergence of novel communicable diseases. Rapid and accurate diagnoses of infectious diseases are essential to preventing the transmission of infectious diseases. Although some commonly used diagnostic technologies provide highly sensitive and specific measurements, limitations including the requirement for complex equipment/infrastructure and refrigeration, the need for trained personnel, long sample processing times, and high cost remain unresolved. To ensure global access to affordable diagnostic methods, loop-mediated isothermal amplification (LAMP) integrated clustered regularly interspaced short palindromic repeat (CRISPR) based pathogen detection has emerged as a promising technology. Here, LAMP-integrated CRISPR-based nucleic acid detection methods are discussed in point-of-care (PoC) pathogen detection platforms, and current limitations and future directions are also identified.

## 1 Introduction

Infectious diseases impose a heavy burden on developing nations, leading to a high number of deaths and long-term disability.^1^ The annual number of deaths has been estimated as 14.9 million globally, accounting for more than 25% of all deaths.^2^ Biological, environmental, and social conditions across the globe have contributed to the emergence and spread of infectious diseases. Moreover, climate change, expanding vector habitats, contact with wildlife, environmental contamination, increased population density, globalization, poverty, and social inequality continue to drive the emergence of outbreaks.^3^ An analysis of the emergence of infectious diseases between 1940 and 2004 has demonstrated that the majority of research and development (R&D) efforts and resource allocation for infectious disease prevention and surveillance has been centered in developed countries.^4^ However, risk maps for zoonotic pathogen emergence have predicted that, contrary to resource allocation trends, wildlife- or vector-borne pathogens are more concentrated in developing countries, suggesting that such global resource allocation may hinder proactive strategies to prevent the spread of pathogens at the early stages of disease emergence and thereby create an imminent threat to global health.

The lack of infrastructure and access to healthcare has rendered point of care (PoC) testing essential to preventing the rapid spread of infectious diseases globally. Despite the clear correlation between early diagnosis and transmission prevention as well as treatment outcomes, the lack of access to PoC diagnostic tests continues to jeopardize global health. Moreover, the development of PoC tests for developing countries has remained necessary yet challenging, as such endeavors require long-term and sustained investments.^5^ Following the “ASSURED” criteria set by the World Health Organization (WHO), the optimization of PoC device parameters of (1) affordability, (2) sensitivity, (3) specificity, (4) user-friendliness, (5) rapidness and robustness, (6) equipment-free structure, and (7) delivery^6^ has been attempted through microfluidics,^7^ antibody-based diagnostic methods,^8^ and nucleic acid amplification techniques (NAATs).^9^ Loop-mediated isothermal amplification (LAMP) integrated clustered regularly interspaced short palindromic repeat (CRISPR) has emerged as a promising tool to create assays for infectious diseases in order to develop highly sensitive, specific, low-cost, user-friendly, and versatile diagnostic devices.

Here, the adaptability of LAMP-integrated CRISPR/Cas technology and its applications in diagnostic testing and PoC platform development are discussed. The current challenges and future directions of LAMP-integrated CRISPR/Cas-based PoC diagnostic tests are elucidated.

## 2 Diagnostic Testing for Infectious Diseases

Rapid and accurate diagnoses of infectious diseases are fundamental to prevent transmission. This not only allows infectious diseases to be contained but also helps patients receive a higher quality of care and improved outcomes. Viral outbreaks in the last two decades, including the severe acute respiratory syndrome coronavirus 2 (SARS-CoV-2) pandemic, have exposed the limitations of existing diagnostic technologies.^10^ Time, ease of application, sensitivity, specificity, and ability to distinguish between variants have become some of the most desirable qualities of diagnostic assays. Yet, current strategies fall short on delivering one or more of these parameters, necessitating the development of improved clinical and PoC testing platforms. To diagnose viral infections, clinical samples are typically tested through NAATs, serological testing to detect viral antigens or specific antibodies, viral cytopathology, or immunofluorescence.^11^ Similarly, bacterial infections can be detected using NAATs, serological testing, immunoassays, cytopathology, antimicrobial susceptibility tests, or cell cultures.^12^ While each strategy has its merits, some, including viral cytopathology and immunofluorescence, require multiple processing steps before specimens can be analyzed and therefore are not ideal for PoC testing purposes. Other strategies, including serological testing, cell cultures, and cytopathology, require trained technicians to collect samples and/or identify cellular morphology and take a long time (up to days/weeks for culturing), proving insufficient for PoC applications.^13^

NAATs have proved to be advantageous over other techniques, as they are highly sensitive and specific and allow for automation. Therefore, they are commonly used to detect foreign genomic material in clinical samples. Commonly used NAATs include the gold-standard polymerase chain reaction (PCR), which enables experimental clinic decision making to identify previously sequenced pathogens. However, it requires significant laboratory infrastructure (i.e., a thermocycler), is expensive, and possesses the risk of sample contamination.^14^ Due to the thermal cycling steps, PCR cannot be employed in low-resource settings and has limited use in PoC platforms. Isothermal amplification strategies including LAMP, recombinase polymerase amplification (RPA), rolling circle amplification (RCA), and nucleic acid sequence-based amplification (NASBA) have been proposed as promising methods to overcome the limitations associated with thermal cycling requirements (Table 1).^15^ Among these methods, RCA remains limited in its application, as it only works with circular templates and requires multiple rounds of amplification, although its working mechanism and primer design are facile. NASBA is designed to detect RNA and produce results rapidly. However, it requires a denaturation step and is efficient in amplifying only a limited range (120–250 bp) of targets. While RPA has a simple primer design and can produce results rapidly with high sensitivity, nonspecific background amplification and primer–primer annealing remain the main challenges. Similarly, although LAMP is a highly sensitive and rapid strategy, primer–primer annealing can be observed, leading to false positive results. Consequently, no stand-alone nucleic acid amplification strategy fulfills all PoC test parameters, warranting novel sensing approaches.

**Table 1:** Brief Summary of the Advantages and Limitations of Isothermal Nucleic Acid Amplification Methods

isothermal amplification method	advantages	limitations	ref
LAMP	65 °C	complex primer design	(16−18)
	robust amplification	potential nonspecific amplification	
	high specificity	false positives	
RPA	37–42 °C	potential nonspecific amplification	(18,19)
	rapid amplification	false positives	
	minimal equipment required		
RCA	23–60 °C	circular templates required	(18,20)
		simple primer design	
NASBA	41 °C	requires denaturation step	(18,21,22)
	rapid result production	limited range of targets (150–250 bp)	
	no DNA strand displacement	higher cost	

## 3 LAMP-Integrated CRISPR/Cas-Based Diagnostic Tools

### 3.1 Loop-Mediated Isothermal Amplification (LAMP)

LAMP-integrated CRISPR/Cas-based diagnostic methods have emerged as promising platforms to overcome the aforementioned limitations. LAMP is a NAAT in which six primers targeting eight distinct sequences of a gene and a Bst DNA polymerase enzyme are used to conduct highly specific nucleic acid amplification at a constant temperature (typically 60–65 °C).^23^ The reaction can therefore be carried out on a simple heating block and does not require a high-cost thermocycler. As the LAMP method does not require any complex equipment or trained personnel to conduct elaborate steps, it appears to be a promising approach for PoC applications. Although LAMP has been used for pathogenic nucleic acid detection for two decades, the SARS-CoV-2 pandemic has particularly accelerated research efforts to use modified LAMP as a PoC diagnostic method for pathogen detection. Moreover, reverse transcriptase LAMP (RT-LAMP) has been used for the colorimetric detection of viral SARS-CoV-2 load,^24−26^ enabling the rapid observation and interpretation of test results. In addition to colorimetric change, visual detection can also be achieved turbidimetrically in real time, as the LAMP reaction produces magnesium pyrophosphate (Mg2P2O7) as a byproduct.^27^ Additionally, LAMP diagnostic tests have been integrated in lateral-flow assays (LFAs).^28^ Aside from producing simple result readouts rapidly, LAMP allows the amplification of a target gene sequence in less than an hour in a single tube, providing ease of application, and offers a higher amplification efficiency than the gold standard, PCR.

While LAMP provides several advantages in pathogen detection over other NAATs, some shortcomings of the strategy have prevented its widespread application. LAMP allows the discrimination of a single nucleotide base difference, yet LAMP primer design is relatively complex.^15^ However, this complexity can lead to an incorrect primer design, thereby causing undesired primer–primer interactions. Furthermore, as a result of inaccurate primer design or indirect detection methods, false positives have been observed. The generation of false positive results has remained a major shortcoming of LAMP. Several strategies have been employed to overcome this challenge. These strategies have included the use of quenched fluorescent primers,^29^ the combination of LAMP with Förster resonance energy transfer (FRET) probes,^30^ and the integration of LAMP into CRISPR-based pathogen detection platforms. Another challenge associated with LAMP-based pathogen detection techniques is the false negative rate (FNR). In particular, for single-stranded RNA viruses, high mutation rates have been observed.^31^ As such, rapidly mutating RNA viruses can increase FNR.^32^ A meta-analysis of 9360 suspected cases of SARS-CoV-2 has demonstrated FNRs of 0.06 and 0.12 for RT-PCR and RT-LAMP, respectively. The diagnostic efficacy could be improved using mixed sampling and multiple gene targets for nucleic acid detection instead of single-site sampling and single gene targets.

### 3.2 CRISPR-Based Detection

The characterization of CRISPR and CRISPR-associated (Cas) proteins has led to remarkable advances in biology. Initially defined as a bacterial and archaeal adaptive immune system against foreign genomic material, the programmability of CRISPR systems has been demonstrated to allow for precise and efficient genome editing.^33^ A chimeric *trans*-activating CRISPR RNA (tracrRNA) and CRISPR RNA (crRNA), termed the guide RNA (gRNA), could effectively guide the Cas endonuclease to cleave double-stranded DNA (dsDNA). Depending on their respective genomic architectures, Cas proteins have been categorized into two classes and six subtypes.^34,35^ The effector modules of class I systems are composed of multiple Cas proteins, whereas a single multidomain protein is used in class II systems. The targeted DNA/RNA cleavage activity of Cas proteins have been used for a wide range of applications from the generation of stress-resistant crops^36^ to the development of therapies to cure symptoms of transfusion-dependent β-thalassemia (TDT) and sickle cell disease (SCD)^37^ or the use of chimeric antigen receptor (CAR) T cell therapy to treat various forms of cancer.^38^

The development of viral diagnostic methods using CRISPR technology has appeared as a particularly promising approach as a result of the programmability,^10,39,40^ specificity,^41,42^ and efficiency of Cas proteins.^43^ Several CRISPR/Cas enzymes have been used in conjunction with a multitude of pre-amplification techniques, including PCR,^44^ reverse PCR,^45^ CRISPR-typing PCR (ct-PCR),^46^ RPA,^47^ NASBA,^48^ and LAMP. Furthermore, Cas9 has been employed to detect SARS-CoV-2, influenza A and B, and respiratory syncytial virus in a single assay.^49^ It has also been used to detect Zika virus^48^ and human papillomavirus (HPV) 16 and 18 (HPV16 and HPV18, respectively).^46^ While these methods have highlighted the programmability of Cas9 and its potential to be used in virus detection, the preamplification steps have rendered these diagnostic methods expensive and complex. In contrast to Cas9 proteins, Cas12, Cas13, and Cas14 proteins inherently possess collateral cleavage activity. Moreover, for Cas9 proteins, *cis*-cleavage is observed, meaning that a site-specific cleavage of a DNA strand containing the target sequence takes place. Cas12 and Cas13 systems are associated with *trans*-cleavage activity where nonspecific collateral cleavage occurs.^50^ Upon target recognition, Cas12, Cas13, and Cas14 cleave nontarget single-stranded DNA (ssDNA), single-stranded RNA (ssRNA), and ssDNA molecules, respectively.^51−53^ Thus, by exploiting this collateral cleavage activity of Cas proteins, functionalized reporter nucleic acid molecules can be employed to fabricate PoC diagnostic assays. Moreover, Cas12- and Cas13-based pathogen detection strategies do not require high-cost equipment nor experienced technicians, thereby enabling the generation of less complex, user-friendly, and scalable diagnostic technologies. Despite these advantages, Cas13 reporters are RNA molecules and are inherently unstable and prone to cleavage by RNases in pathogen detection.^54^ Additionally, stand-alone CRISPR diagnostics provide qualitative results, impeding efforts to monitor viral load in clinical samples.^55^

### 3.3 LAMP-Integrated CRISPR

**Figure 1:**
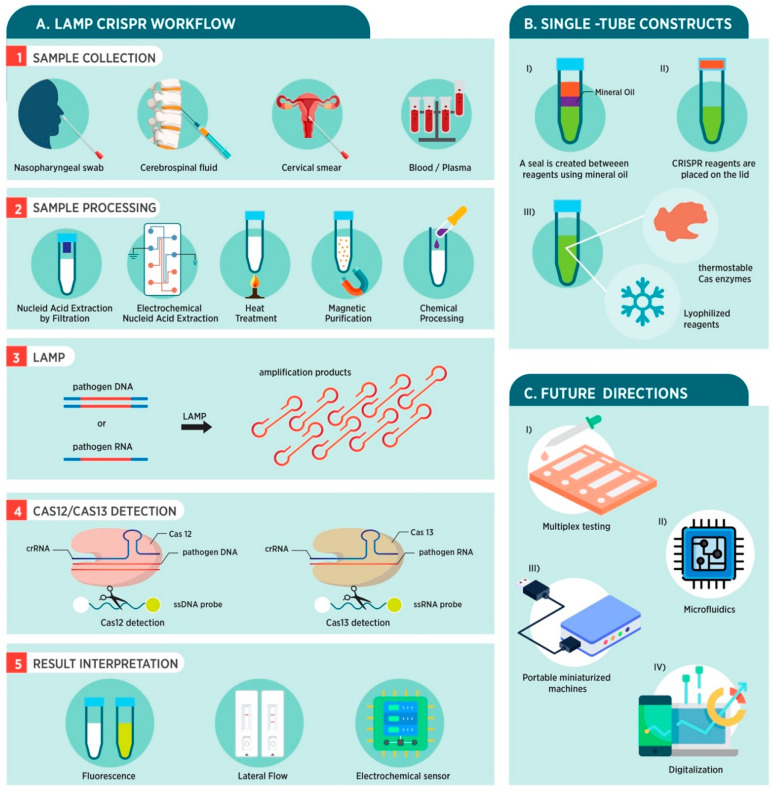
Overview of a conventional LAMP-integrated CRISPR workflow for PoC platforms. (A) The LAMP-integrated CRISPR-based diagnostic mechanism consists of (i) sample collection; (ii) sample preparation; (iii) loop-mediated isothermal amplification at 60–65 °C; (iv) CRISPR-based detection, leveraging the collateral cleavage activity of Cas12 and Cas13 to cleave nontarget nucleic acid molecules upon target recognition; and (v) interpretation of results using fluorescence or LFA. (B) Single-tube platforms are used to reduce contamination, simplify the process for untrained users, and streamline testing. Common strategies to develop single-tube platforms are as follows: (i) mineral oil is used to prevent LAMP and CRISPR reagents from mixing, (ii) CRISPR reagents are placed on the lid of the tube, and (iii) thermostable Cas enzymes and/or lyophilized reagents are used. (C) Future prospects to enhance the functionality of LAMP-integrated CRISPR-based PoC platforms: (i) multiplex testing capabilities, (ii) the integration of microfluidics, (iii) miniaturization of devices, and (iv) digitalization of platforms. Some of the elements in Figure 1 were designed using resources from freepik.com and flaticon.com.

LAMP-integrated CRISPR-based platforms have been developed as promising PoC diagnostic methods to combat the shortcomings of stand-alone NAATs. These platforms rely on the amplification of specific gene sequences of foreign genomic material using LAMP, followed by the detection of amplification products using Cas proteins as guided by the gRNA targeting the amplified gene region (Figure 1). Specifically, Cas12 and Cas13 are commonly used in conjunction with LAMP owing to their collateral cleavage activity. Cas proteins are activated in samples containing the target gene sequence (the amplification products of LAMP), resulting in the cleavage of nontarget quencher molecules. This collateral cleavage activity is often employed as a simple and effective approach to generate a variety of results through LFAs,^56,57^ fluorescence,^58−60^ and visual monitoring^61^ without high-cost equipment. Thus, the LAMP-integrated CRISPR strategy provides easy result interpretation for nontrained users, rendering it effective for use in PoC settings. The robust LAMP amplification allows for a picomolar limit of detection (LOD), implying that even low sample volumes are sufficient to obtain accurate results.^50^ Twenty-four different LAMP-CRISPR-based PoC platforms employing Cas12 or Cas13 and targeting human pathogens have been reviewed. Among these 24 platforms, 18 methods detected human viral pathogens and 6 methods detected human bacterial pathogens in clinical specimens (Table 2). With tests taking between 10 and 120 min, LAMP-CRISPR platforms proved to be rapid. The sensitivity ranged from 50% to 100%, and the specificity ranged from 67.3% to 100%.

**Table 2:** LAMP -Integrated Cas12 and Cas13 Viral and Bacterial Detection Diagnostic Platforms

method	Cas protein	target pathogen	limit of detection^a^	sensitivity (%)	specificity (%)	assay time (min)	assay analysis	ref
detection of viral pathogens								
STOPCovid.v2	Cas12b	SARS-CoV-2	∼RT-qPCR Ct value of 40.3	93.1	98.5	<60	LFA	(56)
							fluorescence	
iSCAN	Cas12a	SARS-CoV-2	∼RT-qPCR Ct of 15–40	50	100	∼40	fluorescence	(57)
	Cas12b		10 copies per reaction	86	100		LFA	
ITP-CRISPR	Cas12a	SARS-CoV-2	10 copies mL^–1^	90.6	100	30–40	fluorescence	(58)
contamination free SARS-CoV-2 detection	Cas12a	SARS-CoV-2	20 copies per reaction	100	100	40	fluorescence	(59)
SARS-CoV-2/DETECTR	Cas12a	SARS-CoV-2	30 copies μL^–1^	100	100	∼40	fluorescence	(60)
			45 copies μL^–1^					
opvCRISPR	Cas12a	SARS-CoV-2	5 copies	NQ	NQ	45	fluorescence	(64)
CRISPR-SPADE	Cas12b	SARS-CoV-2	12–500 copies μL^–1^	92.8	99.4	10–30	fluorescence	(63)
CRISPR-top	Cas12b	SARS-CoV-2	10 copies per reaction	100	73.1	60	fluorescence	(65)
					67.3		LFA	
CLEVER assay	Cas12	SARS-CoV-2	Ct < 33	89.6	100	85	LFA	(66)
SARS-CoV-2 DETECTR	Cas12a	SARS-CoV-2	10 copies μL^–1^	95	100	30–40	LFA	(67)
LAMP-LbCas12a Method	Cas12a	SARS-CoV-2	16 copies μL^–1^	90	100	40	fluorescence	(68)
portable RT-LAMP/CRISPR	Cas12a	SARS-CoV-2	35 copies μL^–1^	NQ	NQ	35	fluorescence	(69)
							LFA	
RT-LAMP-CRISPR	Cas13a	SARS-CoV-2	Ct < 20	83	100	80	LFA	(70)
lab-on-a-chip	Cas12a	SARS-CoV-2	2.3 copies μL^–1^	100	100	120	electrochemical	(72)
UCLD	Cas12a	SARS-CoV-2	10 copies μL^–1^	Up to 83.33	100	115	fluorescence	(71)
		IAV	10 copies μL^–1^					
		IBV	102 copies μL^–1^					
		RSVA	102 copies μL^–1^					
		RSVB	102 copies μL^–1^					
CRISPR/Cas 12a Based Detection	Cas12a	IAV	1 PFU per reaction	NQ	NQ	75–85	fluorescence	(73)
		IBV					LFA	
CIALFB	Cas12a	HPV16	3.1 attomoles	100	100	60	LFA	(28)
		HPV 18						
RAVI-CRISPR	Cas12a	JEV	8.97 copies	NQ	NQ	60	fluorescence	(76)
							LFA	
RT-LAMP Coupled CRISPR/Cas12	Cas12a	HCV	10 ng μL^–1^	96	100	60	fluorescence	(78)
						90	LFA	
detection of bacterial pathogens								
CRISPR/Cas12a-E-LAMP	Cas12a	*Shigella flexneri*	4 copies uL-1	NQ	NQ	40	fluorescence	(61)
CRISPR/Cas12a-LAMP	Cas12a	*Neisseria meningitidis*	40 copies per reaction	NQ	NQ	<120	fluorescence	(81)
CRISPR/Cas12a-LAMP	Cas12a	*Klebsiella pneumonia carbapenemase*	3 × 105 CFU/mL	NQ	NQ	30–40	LFA	(83)
CRISPR-top	Cas12b	*Klebsiella pneumonia*	1.6 × 105 CFU mL^–1^	96	100	60	fluorescence	(84)
							LFA	
LACD	Cas12a	*Myobacterium Tuberculosis*	10 copies per reaction or 50 fg of genomic DNA	79.5	100	60	fluorescence	(85)
							LFA	
TB-QUICK	Cas12b	*Myobacterium Tuberculosis*	1.3 copy μL^–1^ at 95% positive rate	86.8	95.2	120	fluorescence	(87)

a Gold-standard CDC RT-qPCR LoD: five copies per reaction. NQ: not quantified. SARS-CoV-2: severe acute respiratory syndrome coronavirus 2. IAV: influenza A virus. IBV: influenza B virus. RSVA: respiratory syncytial virus A. RSVB: respiratory syncytial virus B. HPV: human papillomavirus. JEV: Japanese encephalitis virus. HCV: hepatitis C virus.

## 4 Detection of Pathogens

### 4.1 SARS-CoV-2

Methods such as specific high-sensitivity enzymatic reporter unlocking (SHERLOCK) have been used to construct highly sensitive, specific, programmable, and rapid platforms for viral pathogen detection.^62^ However, the traditional SHERLOCK method requires a preliminary target amplification process using PCR or other preamplification steps preceding Cas-mediated detection. The method, therefore, remains complex for PoC applications in resource-poor settings. To overcome these limitations, a new method termed SHERLOCK testing in one pot (STOP) was developed, eliminating the requirement for thermal cycling, sophisticated equipment, and trained personnel.^56^ Amplification of the nucleocapsid protein, N, of SARS-CoV-2 was achieved using RT-LAMP at 55–70 °C. Thermostable Cas12b was then used for the detection of the target viral genome, and results were analyzed using LFA or fluorescence. To further increase the sensitivity, a magnetic bead purification step was introduced, reducing the duration of the extraction procedure. With 93.1% sensitivity and 98.5% specificity, STOPCovid.v2 proved to be highly accurate and comparable to the gold-standard RT-qPCR tests used for SARS-CoV-2 detection.

**Figure 2:**
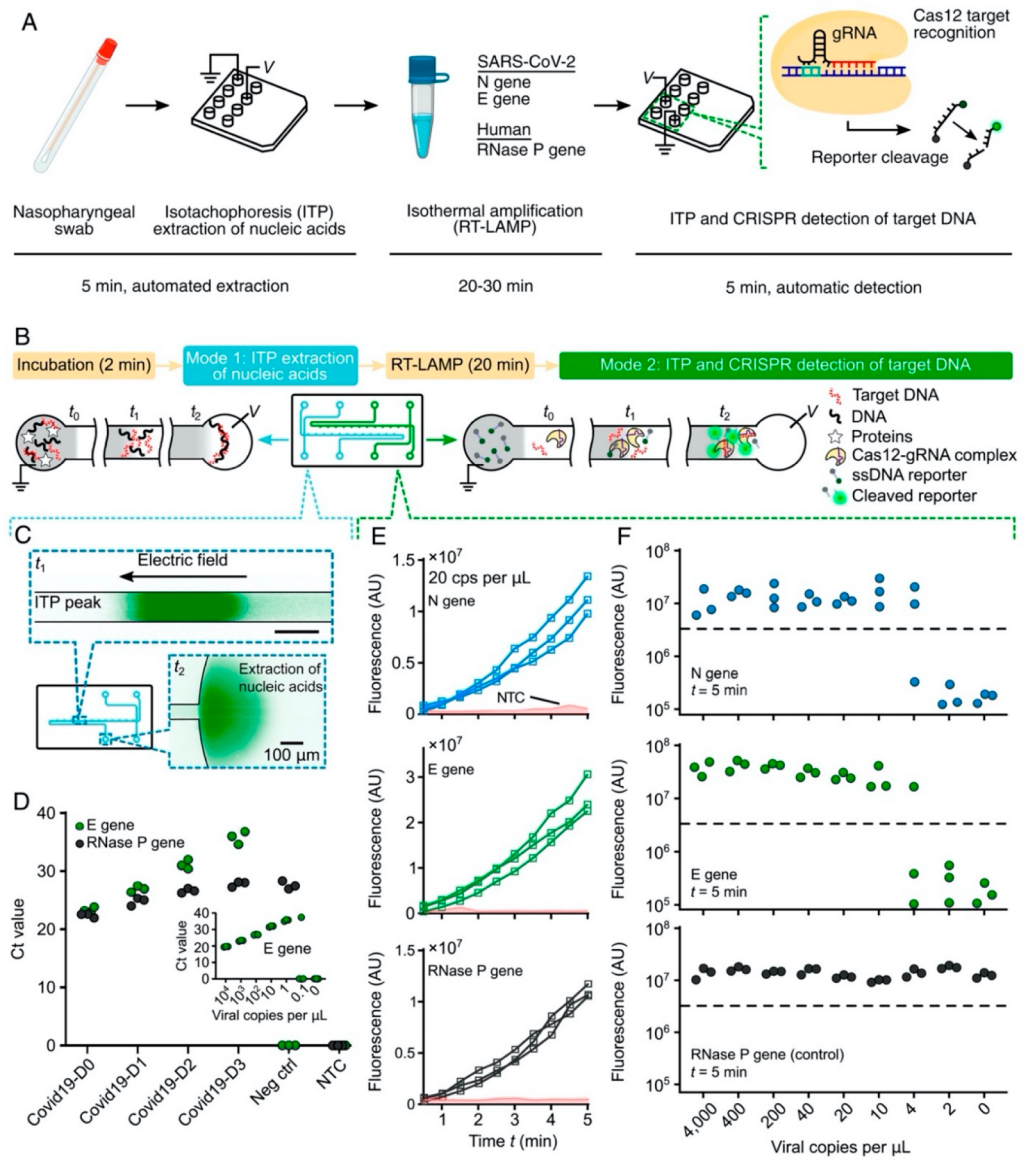
ITP-enhanced CRISPR detection of SARS-CoV-2 in nasopharyngeal swab samples. (A) ITP-CRISPR workflow for SARS-CoV-2 detection. (B) Stepwise schematic of incubation, RNA extraction, RT-LAMP, and ITP-CRISPR detection phases. (C) Nucleic acid extraction from a nasopharyngeal swab sample on chip. (D) RT-PCR of ITP-extracted nucleic acid samples obtained from clinical nasopharyngeal samples. D0–D3 Covid-19 samples are used as negative controls. D1–D3 Covid-19 samples are 1:10 serial dilutions of the D0 Covid-19 sample. (E) Fluorescence signals obtained for N, E, and RNase P gene amplification products following RT-LAMP. (F) Analytical LOD for ITP-CRISPR detection. Adapted from ref (58) in accordance with the Creative Commons Attribution License 4.0 (CC BY).

The platform was termed isotachophoresis (ITP) enhanced CRISPR (ITP-CRISPR) and was designed to detect SARS-CoV-2 using an electric field-driven ionic focusing technique, ITP, for purification, LAMP for amplification, and Cas12a for detection (Figure 2).^58^ ITP was implemented on a microfluidic chip and served to concentrate and cofocus Cas12-gRNA, target DNA, and reporter ssDNA. Following this step, RT-LAMP was designed to target the N and E genes of viral RNA and the human RNase P gene as a control. Fluorescent signals for positive samples were observed in approximately 3 min, and the assay took between 30 and 40 min to complete, offering a rapid alternative to commonly used viral detection tests. Using less than 0.2 μL of reagents, the microchip-based platform was cost-effective compared to conventional tests, which use up to 100 μL of reagents. Although manual steps required for lysis and LAMP reactions and 10 μL of raw sample remain limitations, the sensitivity (∼90%), specificity (100%), rapidity, and minimal reagent use of the ITP-CRISPR platform demonstrate its suitability for PoC diagnostic applications.

Single-tube testing platforms for PoC applications have gained momentum, as they reduce the risk of contamination, streamline the assay, and simplify the process for untrained workers. To fabricate single-tube platforms, various strategies were employed (Figure 1B). Mineral oil was used to separate reagents by creating a seal;^59^ some reagents were placed on the lid, while others were placed inside the tube;^60^ and an in-house multiplexing device was used to ensure one-pot diagnostics.^63^ A major challenge of single-tube testing is to ensure that reagents for both LAMP and CRISPR remain stable at the same temperature. Thus, utilization of thermostable Cas enzymes, like *Alicyclobacillus acidiphilus* Cas12b (AapCas12b), is crucial as the LAMP reaction occurs at 60–65 °C. Nevertheless, AapCas12b collateral cleavage activity decreases over 60 °C, thereby leading to slower diagnostic performance, as demonstrated by the STOPCovid platform.^56^ Lyophilized reagents and dual orthogonal fluorescence channels, as well as the uniquely thermostable *Brevibacillus sp*. Cas12b (BrCas12b), could render the assays more stable.^63^

A contamination-free SARS-CoV-2 detection method was developed for use in point-of-care settings using LAMP and Cas12a.^59^ Furthermore, RT-LAMP reagents and extracted RNA were added to a tube and covered with mineral oil to prevent volatilization and contamination. The walls of the tube were embedded with the reagents required for the Cas12a-based detection reaction to proceed. The open reading frame (ORF), N, and E genes of SARS-CoV-2 were amplified using RT-LAMP prior to detection. Common respiratory viruses, including influenza virus A and B (FluA and FluB, respectively); coronavirus 229E, NL63, OC43, and HKU1; human adenovirus; human respiratory syncytial virus (RSV-A and RSV-B); human parainfluenza virus (PIV; PIV2–4); human metapneumovirus (hMPV); human bocavirus; human enterovirus; human rhinovirus; and polyomavirus (PyV) were tested to validate the specificity of the method. Only the ORF gene produced fluorescence, indicating a high specificity. Moreover, results were analyzed either by a smartphone or eye and demonstrated 100% agreement with seven positive and three negative samples diagnosed by traditional methods. Complicated infrastructure or high-cost equipment were not required. The assay took 40 min to complete. While the sample size was limited, analysis of the accuracy, specificity, speed of reaction, and ease of results revealed the potential applications of this platform for PoC settings.

**Figure 3:**
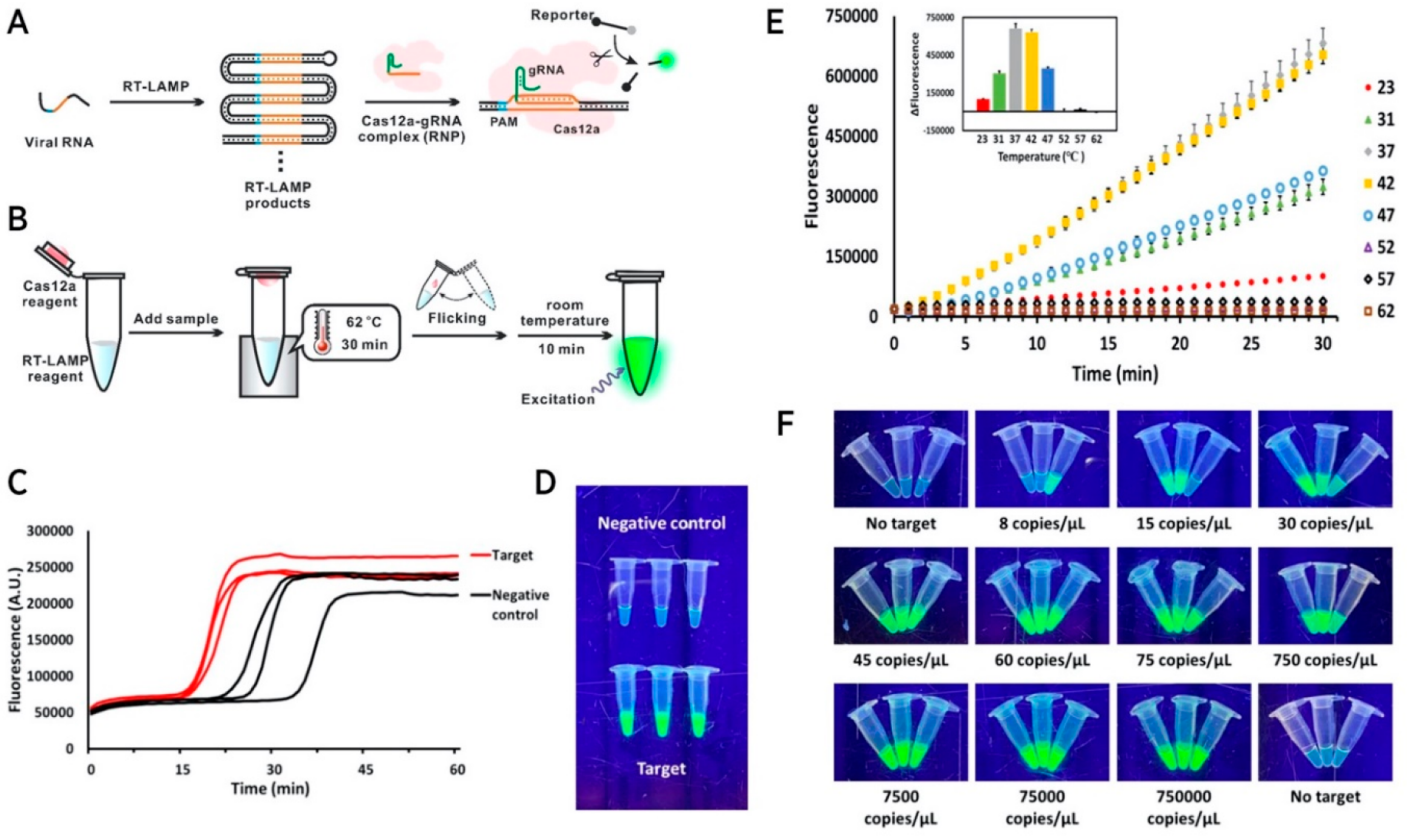
Single-tube RT-LAMP-integrated with CRISPR to detect SARS-CoV-2. (A) Working mechanism of RT-LAMP and CRISPR. (B) Schematic of the single-tube setup. (C) Fluorescence measurement depicting RT-LAMP amplification curves of the target N gene of SARS-CoV-2 and negative controls. (D) Cas12a-based detection of RT-LAMP amplification products. (E) Fluorescence of Cas12a-based detection at different temperatures. (F) Detection of the N gene at a range of concentrations (0–750 000 copies μL^–1^) of 5 μL of sample. Adapted from ref (60) in accordance with the Creative Commons CC-BY-NC-ND license.

Other methods for SARS-CoV-2 detection have been developed utilizing various modifications to optimize sensitivity and specificity or to minimize the time and/or equipment requirement. To develop a single-tube detection platform for SARS-CoV-2 and to produce a readout in 40 min, RT-LAMP-incorporated Cas12a was employed (Figure 3).^60^ By adding LAMP amplification reagents targeting N and E gene regions into the tube and the Cas12a detection reagents into the lid, 100% sensitivity and 100% specificity were achieved when 100 human respiratory swab samples were tested. Positive samples a showed green fluorescent signal, and results were analyzed using a smartphone camera. Another single-tube SARS-CoV-2 detection platform, termed one-pot visual RT-LAMP CRISPR (opvCRISPR), was generated by sealing LAMP amplification reagents with 25 μL of mineral oil, and Cas12a detection reagents were added to the lid.^64^ The opvCRISPR platform produced 100% agreement with results obtained from traditional PCR methods when 24 infected and 26 uninfected clinical samples were tested. Another platform offering single-tube SARS-CoV-2 detection was established using RT-LAMP and Cas12b.^63^ The platform, termed the CRISPR single-pot assay for detecting emerging variants of concern (CRISPR-SPADE) demonstrated high sensitivity (92.8%) and specificity (99.4%) in detecting alpha (B.1.1.7), beta (B.1.351), gamma (P.1), delta (B.1.617.2), and omicron (B.1.1.529) variants. Similarly, using Cas12b-integrated LAMP, CRISPR-mediated testing in one pot (CRISPR-top) was developed to detect SARS-CoV-2 in resource-poor settings, yielding similar diagnostic accuracy compared with the gold standard, RT-PCR.^65^

To overcome the issue of contamination during nucleic acid extraction, a platform termed CRISPR-Cas-integrated RT-LAMP easy, visual and extraction-free RNA (CLEVER) was developed.^66^ Using RT-LAMP to amplify the N and E genes of SARS-CoV-2 along with an internal control POP7 gene and Cas12 for detection, the CLEVER method produced a LFA readout in 85–100 min. With a sensitivity of 89.6% and specificity of 100%, the assay offered a promising low-cost solution for SARS-CoV-2 detection, particularly in resource-poor settings. Another RT-LAMP-integrated Cas12a-based platform targeting the E and N genes had 95% positive agreement and 100% negative agreement when 36 positive and 42 negative human clinical samples, which were confirmed using qRT-PCR, were tested.^67^ LFA readouts were produced in under 40 min, and the CRISPR-based DETECTR technology could be reconfigured within days, offering significant advantages for the rapid generation of pathogen detection technologies.

Alternatively, other platforms have been developed integrating RT-LAMP amplification and Cas12a-based detection following a low-cost and rapid (25 min) RNA isolation step.^68^ Various other modifications, including the development of a portable RT-LAMP/CRISPR machine^69^ and the integration of Cas13,^70^ have been established to ensure SARS-CoV-2 detection in PoC settings. To achieve more sensitive results, uracil-DNA-glycosylase (UDG)-RT-LAMP was used in combination with Cas12a, leading to fewer false positive results and increased point-mutation detection efficiency for SARS-CoV-2.^71^

While most LAMP-integrated CRISPR-based strategies have focused solely on the identification of viral genomes in human clinical samples, a lab-on-a-chip platform was developed to detect both SARS-CoV-2 RNA and anti-SARS-CoV-2 antibodies in saliva and plasma samples.^72^ Purification steps prior to amplification and detection were eliminated to allow for the use of untreated saliva for on-site applications. Viral RNA and host antibodies were detected on the same electrochemical (EC) chip. The compact design incorporated two high-power resistors as heating elements and four chambers, a 50 μL reaction chamber that contains a poly(ether sulfone) (PES) membrane, a 25 μL LAMP reservoir, a 22 μL CRISPR reservoir, and a 20 μL reservoir for direct saliva-based antibody detection. Cas12a targeted highly conserved regions of the SARS-CoV-2 genome, such as the open reading frame (ORF1). With 100% sensitivity and specificity in detecting SARS-CoV-2 specific IgG and viral RNA simultaneously using a multiplex EC sensor, the platform had promising results for PoC applications.

### 4.2 Other Viral Pathogens

To rapidly detect and prevent the spread of influenza viruses A (IAV) and B (IBV) on-site, a RT-LAMP-mediated Cas12a-based assay was developed.^73^ The IAV matrix (M) and IBV hemagglutinin (HA) genes were amplified using two methods: RT-RPA and RT-LAMP. Upon amplification, amplicons were detected using a DETECTR assay, where Cas12a, IAV M gene- and IBV HA gene-targeting gRNAs, and a ssDNA-fluorophore (FAM) quencher (FQ)-labeled reporter were employed. Although nonspecific products of RT-RPA were detected, only target products were detected for the RT-LAMP method. Cross-reactivity was not observed for either method. High specificity and sensitivity were reported at one plaque-forming unit (PFU) per reaction prepared samples. However, inhibitors in saliva decreased the sensitivity of the assay 10-fold, showing limitations for use in PoC settings. While results could be acquired using both fluorescence and LFAs, LFA resulted in the formation of a positive band if the reaction time was over two min, resulting in false positives.

HPV16 and HPV18 remain the most common causes of cervical cancer in women.^74^ To detect HPV16 and HPV18 accurately and rapidly, a high-fidelity LAMP-integrated Cas12a-based LFA biosensor, termed CIALFB, was developed.^28^ LAMP was used to amplify a hypervariable loop V of a conserved region L1 gene on the HPV16 and HPV18 genomes. Upon recognition of the amplification products, Cas12a *trans*-cleavage of an ssDNA reporter occurs. Uncleaved reporters could hybridize their complementary test line probes, producing a line for HPV16 and HPV18 negative samples, whereas cleaved reporters were unable to hybridize the test line, rendering the test line undetectable. LFA readout would therefore produce an undetectable test line for positive samples. Furthermore, in 60 min, no test line was observed for positive samples containing a concentration as low as 3.1 × 10–18 M (1.8 copies in 2 μL) of HPV16 or HPV18, demonstrating the sensitivity of CIALFB. Fourteen cervical samples (seven positive and seven negative confirmed using PCR) were tested using CIALFB, and the results demonstrated 100% accuracy. Moreover, with its high efficiency, sensitivity, and cost-effectiveness, the platform offered an alternative to traditional methods used in clinical settings.

Japanese encephalitis virus (JEV) is an endemic flavivirus that spreads through mosquitos, and a JEV infection can lead to inflammation of the brain, i.e., encephalitis.^75^ A rapid visual CRISPR assay termed RAVI-CRISPR was developed to ensure accurate, sensitive, and convenient diagnosis of JEV.^76^ RT-LAMP was used to amplify the JEV C gene, and five CRISPR RNAs (crRNAs) targeting the C gene were designed. Further experiments to assess sensitivity and validate the specificity of the platform were conducted using crRNA producing the highest fluorescence signal intensity. Ease of application was achieved by a single-tube reaction. Moreover, the RT-LAMP mixture, an insulating layer composed of mineral oil, was added to the bottom of the PCR tubes. With the LAMP mixture layer was gently mixed 1 μL of RNA, and the sample was incubated at 65 °C for 40 min to ensure sufficient time for amplification. The mixture was then vortexed, thereby initiating the Cas12a-based detection step. Upon target binding of Cas12a, ROX-dye ssDNA-FQ reporter quenching was initiated, producing a fluorescence signal visible to the eye. As porcine viruses are common in swine production systems and can cause mixed infections with JEV, the specificity of the assay was evaluated using RNA extracted from porcine reproductive and respiratory syndrome virus (PRRSV), porcine delta coronavirus (PDCoV), transmissible gastroenteritis virus (TGEV), and porcine epidemic diarrhea virus (PEDV), along with a plasmid containing the African swine fever virus (ASFV) P72 gene. Cross-reactivity with other porcine viruses was not observed, indicating that the single-tube platform was highly specific to JEV. Although human clinical samples were not tested, validation was achieved using JEV-infected mice brain samples. RAVI-CRISPR results demonstrated 100% agreement with results obtained using RT-qPCR. Thus, RAVI-CRISPR offered a low-cost and convenient method for PoC diagnostic applications.

**Figure 4:**
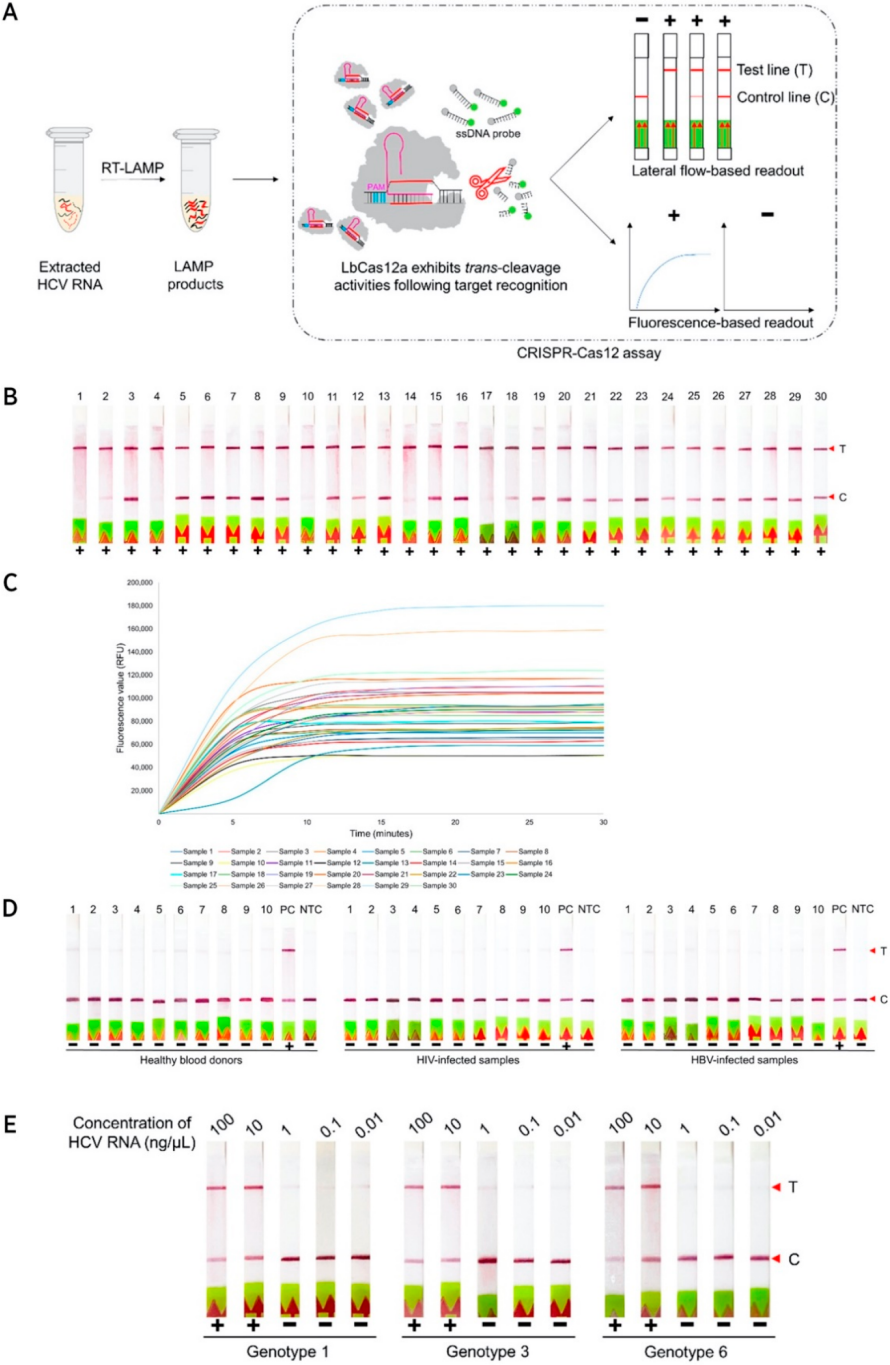
HCV detection in human plasma samples using RT-LAMP and CRISPR. (A) Schematic of the RT-LAMP and CRISPR-based platform to detect HCV. (B) LFA readout for clinical samples. (C) Fluorescence-based readout for clinical samples. (D) LFA readout of RT-LAMP-coupled CRISPR detection of HCV for 10 healthy blood donor samples, 10 HIV-infected clinical samples, and 10 HBV-infected clinical samples. (E) LOD detection of HCV genotypes 1,3, and 6 at a range of concentrations (0.01–100 ng μL^–1^). Adapted from ref.^78^ in accordance with the Creative Commons CC BY license.

Hepatitis C virus (HCV) can cause liver inflammation and lead to severe liver damage, including liver cirrhosis and hepatocellular carcinoma.^77^ The diagnosis of HCV in resource-limited settings has remained challenging as a result of the high-cost equipment requirements of conventional tests. To address this challenge, an RT-LAMP-integrated Cas12a-based biosensor was constructed to increase the availability of molecular HCV testing in resource-poor settings (Figure 4).^78^ Following RNA extraction, the viral RNA was amplified using RT-LAMP. A crRNA targeting the 5′-noncoding region (NCR) of HCV subtypes was designed for Cas12a activation. A fluorescence signal detector was used to assess the Cas12a detection results. Additionally, an LFA was constructed in which a control band would disappear for positive samples as a consequence of Cas12a *trans*-cleavage activity. Results showed faint or absent bands for positive samples tested using the LFA after 30 min of incubation. 96% agreement was acquired for 100 RT-PCR-confirmed positive samples. Out of 10 HIV infected, 10 HBV infected, and 10 healthy plasma samples, none showed positive results when tested using LFA and fluorescence. While not all HCV subtypes were tested and RT-LAMP inhibition was not assessed, the short readout time, high sensitivity, high specificity, disposability, and suitability for large-scale production of the platform offer significant advantages for PoC settings.

### 4.3 Detection of Bacterial Pathogens

While bacterial culture from human saliva, blood, or cerebrospinal fluid (CSF) is often used for accurate bacterial infection diagnosis, culturing does not provide rapid results; the patient and attending healthcare workers typically must wait several days before diagnosis is confirmed. Therefore, the rapid detection of bacterial pathogens in patient samples is crucial to ensure timely treatment. LAMP-integrated Cas12 platforms have been used not only for the successful detection of viral pathogens but also to detect bacterial pathogens. *Shigella flexneri*, a human pathogen causing diarrhea, remains and the most common cause of bacillary dysentery and negatively impacts patients worldwide, particularly in developing countries.^79^ To provide a rapid and accurate platform for *S. flexneri* detection, a Cas12a enhanced loop-mediated isothermal amplification (Cas12a-E-LAMP) platform was developed.^61^ A highly conserved sequence, a hypothetical protein gene, was amplified using LAMP and then covered with mineral oil to prevent contamination. The detection of results was achieved using the fluorescence signal generated by the Cas12a *trans*-cleavage products. Results could be observed by eye, offering ease of use for resource-poor as well as PoC settings. Moreover, the sensitivity and specificity of the platform were validated by comparing results obtained from *S. flexneri* samples to those from other bacteria, including *Enterococcus faecalis, Salmonella enteritidis, Klebsiella pneumoniae, Proteus mirabilis, Escherichia coli*, and *Staphylococcus aureus*, and a fluorescence signal was only observed for *S. flexneri* samples, indicating high sensitivity. The limit of detection (4 copies μL^–1^) of the Cas12a-E-LAMP platform was comparable to those of LAMP and qPCR and 10-fold higher than that of PCR, demonstrating high sensitivity.

Without rapid detection and antibiotic administration, bacterial meningitis caused by *Neisseria meningitidis* can result in sepsis and death.^80^ To develop a rapid and affordable *N. meningitidis* detection platform that could operate in low-resource settings, a LAMP-integrated Cas12a-based method was employed.^81^ The MetA gene of *N. meningitidis* was amplified using LAMP. Cas12a was then utilized to avoid false-positive results, a problem often encountered when using standalone amplification-based detection methods such as PCR. Upon amplification, a fluorescent reporter was cleaved as a result of the collateral cleavage activity of Cas12a in *N. meningitidis*-positive samples, and fluorescence was observed even at low concentrations (40 copies of *N. meningitidis*). Validation of the method was achieved by testing 51 cerebrospinal CSF samples obtained from patients suspected of experiencing bacterial meningitis. While 18 of the 51 samples tested positive for *N. meningitidis* using PCR, 13 of the 51 samples tested positive when the LAMP-Cas12a method was employed. Importantly, the 13 samples identified as positive result by LAMP-Cas12a were not only identified as positive result using PCR but also matched the patients’ clinical symptoms. The additional 5 samples identified as positive results using PCR did not match the patients’ clinical symptoms, indicating that the PCR test resulted in false positives. Moreover, results demonstrated the accuracy of the platform, suggesting its use in clinical settings to achieve higher sensitivity and specificity for bacterial meningitis detection.

**Figure 5:**
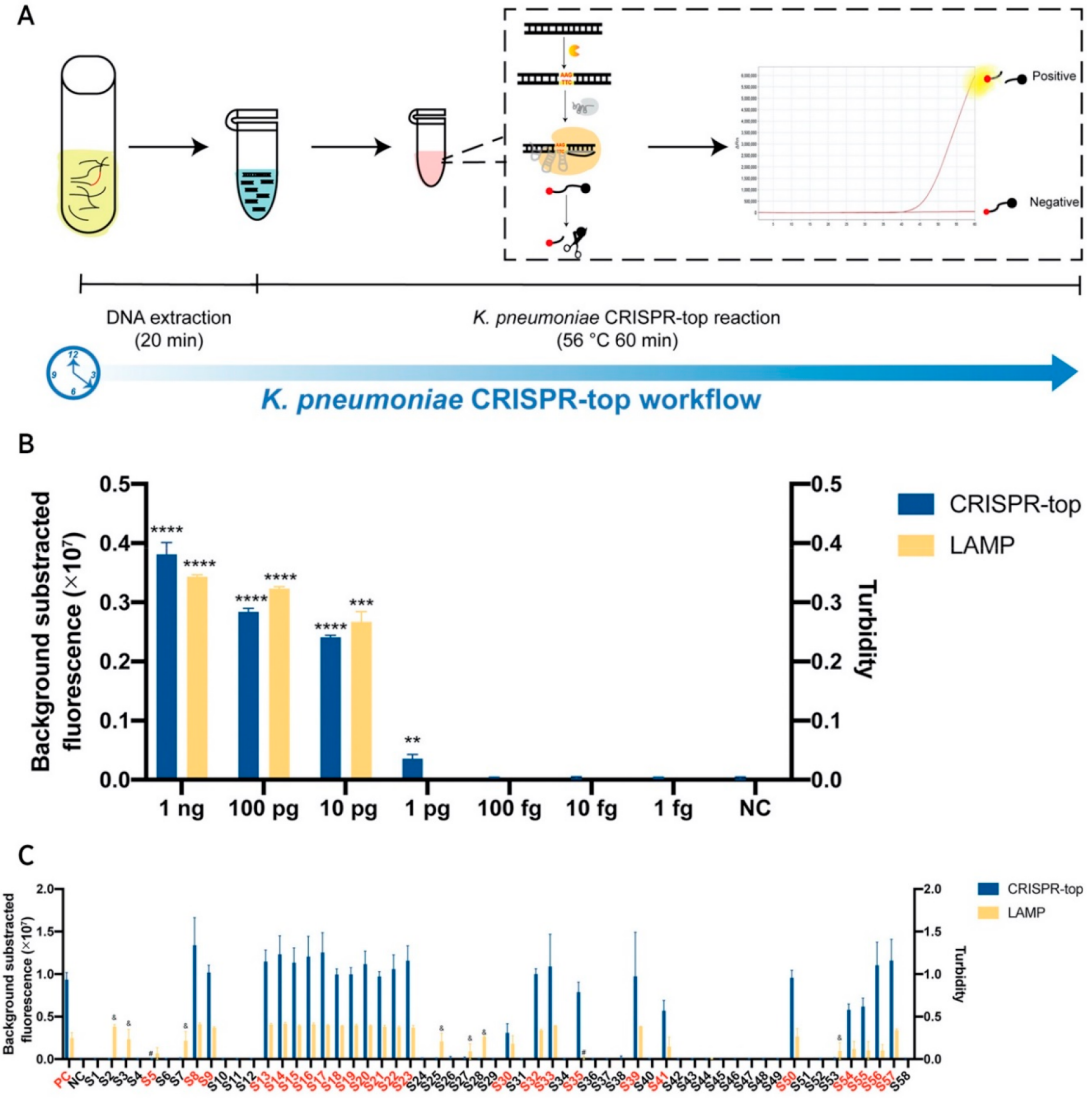
Single-tube LAMP-integrated CRISPR assay to detect *K. Pneumoniae* in clinically isolated sputum samples. (A) CRISPR-top workflow for DNA extraction, LAMP amplification, and CRISPR detection of *K. pneumoniae* in a single tube. (B) Analytical LOD of CRISPR-top and standalone LAMP. (C) Validation of CRISPR-top and standalone LAMP in clinical samples. Red indicates samples with positive results for *K. pneumoniae*. PC = positive control (*K. pneumoniae* ATCC 700603), NC = negative control, & = false-positive results, and # = false-negative results. Adapted from ref (84) in accordance with the Creative Commons Attribution 4.0 International license.

Antibiotic resistant bacterial infections, particularly in hospitals, have been a growing health issue across the globe. The emergence of *Klebsiella pneumoniae carbapenemas*e (KPC) has led to the generation and spread of KPC-producing enterobacterales.^82^ While currently used diagnostic assays for KPC are considered reliable, they have remained expensive and time-consuming and have produced high false positive result rates. To overcome these limitations, a CRISPR/Cas12a-LAMP assay was developed.^83^ Genes encoding for the KPC and New Delhi metallo-β-lactamase (NDM) were amplified using LAMP. The *trans*-cleavage activity of Cas12a was then utilized for detection by cleaving 5′-FAM-flourescein-3′-BHQ double-labeled reporters, producing fluorescence signal changes for positive samples. An immunochromatographic strip was also developed to ensure a simple application for PoC settings. The reporters used in the Cas12a assay were specifically designed to bind to the immunochromatographic strip when cleaved. Furthermore, an anti-FAM antibody fixed on a test line on the immunochromatographic strip was used to capture the FAM fluorescein cleaved from the reporter, producing a band on the strip. Additionally, a colloidal gold-coupled antimouse antibody was added on a second line on the strip to capture the biotin molecule cleaved from the reporter, producing a secondary band on the strip. With a reporter concentration as low as 50 nm, the lines were visible to the eye, offering an ease of application. Although the kit requires storage at −20 °C and is applicable only to bacterial strains with known genotypes, with a reduced turnaround time of 30–40 min, high accuracy, ease of use, and affordability, the method remains promising for use in PoC settings. Similarly, to detect *K. pneumoniae*, a CRISPR-mediated testing in one pot (CRISPR-top) was developed using LAMP amplification and Cas12b-based detection in a single tube, minimizing fluid handling steps (Figure 5).^84^ 64 *K. pneumoniae* and 41 non-*K. pneumoniae* clinically isolated samples resulted in 100% specificity, and *K. pneumoniae* isolated from 25 sputum samples had 96% sensitivity. With each reaction costing $3.50, the assay offered a rapid solution to *K. pneumoniae* testing.

Another method integrating LAMP-based amplification and Cas12a-based detection was developed to offer a versatile platform for viral or bacterial pathogen detection. The feasibility of the platform termed loop-mediated isothermal amplification coupled with CRISPR-Cas12a-mediated diagnostic (LACD) was validated using samples containing the *Myobacterium tuberculosis* complex (MTC).^85^ LAMP primers were designed to target the MTC IS6110 gene, and the FIP regions on the primers were engineered to contain protospacer adjacent motif (PAM) sites (TTTT). A specific gRNA was also designed to target the MTC IS6110 gene. As a result, Cas12a effectors were activated by the LAMP amplification products containing PAM sites, as well as the MTC IS6110 gene. Once activated, the Cas12a/gRNA complex led to the rapid cleavage of ssDNA reporters, producing fluorescence signal changes. The diagnostic test could be completed in 60 min, and results were analyzed using real-time fluorescent signal data as well as a LFA. With a limit of detection of 50 fg, the platform proved to be highly sensitive. Seventeen samples tested positive, all of which contained bacterial species belonging to MTC, revealing the specificity of the platform. When the adaptability of the platform to clinical settings was tested, the MTC-LACD assay detected 35 positive samples out of 44 specimens. A sensitivity of 79.5% was achieved using the assay, which offered significantly higher accuracy compared to that of conventional cell culture (45.5%).

As *M. tuberculosis* leads to the infectious disease tuberculosis (TB), the rapid and accurate diagnosis of TB in PoC settings remains imperative to the effective interventions at an early stage.^86^ To rapidly detect the H37Rv genome of *M. tuberculosis*, a LAMP-integrated Cas12b-based platform, TB-QUICK was devised.^87^ LAMP was used to amplify the IS6110 gene, and a mixture containing Cas12b, sgRNA targeting IS6110, and a PQ probe was added to the amplification products to detect the presence of *M. tuberculosis* in clinical samples. Fluorescence signals were observed in positive samples upon PQ probe cleavage, and a specificity of 100% was achieved. Furthermore, 10 clinically prevalent nontuberculous mycobacteria (NTM) samples were tested, and none produced positive signals. 68 pulmonary samples from TB-positive patients (before and after treatment) and 21 sputum samples from non-TB respiratory infection patients were collected. For TB-positive samples, a sensitivity of 86.5% was obtained, and for non-TB infection samples a sensitivity of 95.2% was obtained. TB-QUICK proved to be more sensitive than conventional culture methods (66.7%). Additionally, by producing test results in under 2 h, this platform is promising for use in PoC settings for improved accuracy in TB diagnostics. Both platforms, TB-QUICK and LACD, were validated using genomic DNA extracted from patient sputum samples. While TB-QUICK offered a higher sensitivity and a lower detection limit than LACD, the specificity of TB-QUICK was slightly lower (95.2%) than that of LACD (100%). Both tests targeted the same gene region and used variants of Cas12; however, TB-QUICK required 120 min to produce results, while LACD only required 60 min.

## 5 Current Challenges and Future Prospects

LAMP-integrated CRISPR has been employed as a versatile method to detect human pathogens obtained from human respiratory swab samples, blood plasma samples, cervical samples, and CSF. The rapid production of results can provide advantages in the diagnoses of infectious diseases in clinical and PoC settings, improving patient care and treatment outcomes, and can help prevent the transmission of infectious diseases, offering protection at the community and global levels. Alternative strategies, including RPA-integrated CRISPR, have also been used in a similar manner. Moreover, RPA-integrated CRISPR detection platforms for SARS-CoV-2 were constructed and typically included four steps: (i) nasal swab or saliva samples were obtained from patients; (ii) samples were processed using a nucleic acid extraction procedure or a chemical and/heat treatment; (iii) E, N, S, or Orf1ab gene regions of the viral RNA was amplified by RT-RPA; and (iv) viral RNA was detected by Cas12 or Cas13 and results were visualized by LFA or fluorescence.^88−92^ To enhance the sensitivity and specificity of RPA integrated CRISPR-based platforms, several strategies have been employed. l-Proline was demonstrated to increase target amplification of RPA and LAMP as well as Cas12a/Cas13a enzymes.^93^ Alternatively, Cas enzymes were engineered to increase robustness. An *Agrobacterium tumefaciens* protein, VirD2, was fused to a catalytically inactive “dead” Cas9 (dCas9), and a LFA-coupled nucleic acid test (Vigilant) was developed.^94^ Additionally, a single-tube platform incorporating RT-RPA and DETECTR to rapidly diagnose SARS-CoV-2 and influenza H1N1 was developed.^95^ A lab-on-paper platform was fabricated to detect SARS-CoV-2 N, S, and RNase P genes simultaneously,^96^ and an instrument-free microfluidic system that contained all RPA, CRISPR, and LFA components was clinically validated, demonstrating 94.1% sensitivity and 100% specificity for SARS-CoV-2.^97^ Beyond PoC viral disease diagnosis, RPA-integrated CRISPR-based platforms have also been used to diagnose *Bordetella pertussis* at PoC settings using clinical swab samples through a single-step system,^98^ to monitor the presence of pathogens such as *S. aureus*(99) or *Toxoplasma gondii*(100) in the environment, and to detect Brucella in blood and milk samples.^101^

The integration of isothermal nucleic acid amplification (INAA) strategies with CRISPR/Cas enzymes remains promising for PoC infectious disease diagnosis as well as other microorganism monitoring purposes. Furthermore, both RPA-integrated CRISPR and LAMP-integrated CRISPR-based platforms can be modified to target emerging pathogens and/or variants. While the versatility, programmability, robust amplification and rapid detection ability, and ease of application have rendered LAMP-integrated CRISPR a promising method for PoC applications, several challenges should be addressed to ensure the scalability of LAMP-integrated CRISPR-based PoC platforms. In addition to extensive testing for assay validation, future directions should focus on expanding the capabilities of the LAMP-integrated CRISPR platforms. Moreover, the development of highly sensitive and specific multiplex testing is essential to ensure improved diagnostic outcomes and provide convenience for patients, particularly in low-resource settings.^102,103^ Additionally, for pathogens with high mutation rates, achieving single-base resolution might be crucial to distinguish between variants and to avoid FNR.^104^ A shortcoming of LAMP-integrated CRISPR technologies is the lack of the quantification of the pathogen concentration. While not essential for diagnosis, rapid viral load quantification in PoC settings can help guide disease management.

High-throughput and multiplex testing to detect different variants of SARS-CoV-2 has been achieved using LAMP-integrated CRISPR;^63^ however, this platform has not yet been transformed into a portable device. Alternative approaches, particularly the use of stand-alone CRISPR-based platforms, are promising in overcoming this challenge. Multiplex testing that can detect Dengue or Zika virus in patient liquid biopsy samples has been developed using a two-target platform utilizing orthogonal CRISPR enzymes.^105^ Furthermore, multiple-target CRISPR platforms can offer expanded diagnostic capabilities for use in low-resource settings. Another important objective for LAMP-integrated CRISPR platforms is achieving high-throughput testing. While many platforms have demonstrated high sensitivity and high specificity rates, the availability of high-throughput testing remains limited. To ensure that high numbers of samples are tested rapidly or samples are tested for multiple viral or bacterial pathogens simultaneously, platforms should enable the simultaneous processing of multiple specimens. This could be accomplished through the integration of microfluidics or miniaturized devices.^106^ The use of microfluidics and the miniaturization of platforms can also decrease the amounts of reagents needed, thereby decreasing the assay cost.

Although the development of single-tube assays has decreased the risk of contamination significantly and simplified the LAMP and CRISPR reaction procedures for untrained users, most single-pot assays still require a DNA/RNA isolation step or sample preparation steps. These additional steps can increase sensitivity and specificity of the assay, but the trade-off is lower field deployability. Particularly for low-resource settings, it is imperative to minimize the preparation steps without compromising sensitivity and specificity. The minimization of predetection steps would not only eliminate the need for trained personnel but also potentially decrease the time for result readouts. Another important consideration that could enable the widespread use of LAMP-integrated CRISPR-based diagnostic platforms is the streamlining of analysis steps. Furthermore, by utilizing smartphone applications,^59^ the interpretation of results could be simplified for nontrained users. To ensure widespread use of LAMP-integrated CRISPR at PoC settings globally, PoC platforms should also be optimized to offer low-cost, sensitive, and specific tests that minimize sample processing steps to prevent contamination and offer a higher ease of application. To achieve these assay characteristics, strategies including Cas protein engineering, integration of microfluidic systems into PoC devices, miniaturization of machines, and incorporation of machine learning can be employed (Figure 1C).

### 5.1 Engineered Cas Proteins

Cas protein engineering has enabled robust activity,^107^ less off-target cleavage,^108^ and switch-like conditional activity.^109^ Recently, *Leptotrichia wadei* (Lwa)Cas13a was engineered to possess enhanced collateral cleavage activity.^110^ RNA-binding domains (RBDs) were inserted in the active site proximal loop, and collateral cleavage activities were observed. Collateral cleavage activity and stability of Cas13a were increased. Furthermore, RBD-LwaCas13a proteins were used in a U20 RNA redox-reporter-functionalized SPE device, and SARS-CoV-2-positive samples were identified. Notably, the samples were unextracted clinical samples, and preamplification steps were not conducted, demonstrating the efficacy of the engineered Cas13a protein. Furthermore, with an LOD of 0.6 copies μL^–1^ in detecting SARS-CoV-2, the engineered protein proved to be highly sensitive and therefore holds promise for use in nucleic acid detection applications. Using a different approach, a photocontrolled crRNA-activated Cas12a was used to create an RPA-integrated CRISPR platform to detect SARS-CoV-2.^111^ To prevent contamination resulting from multiple-step detection platforms, a strategy was developed to conduct LAMP amplification and CRISPR detection sequentially in a single closed test tube. The temporary silencing of the inactivated crRNA was achieved by using a photocleavable (PC) linker containing a protective nucleic acid. Following the RPA reaction, the crRNA was activated using light irradiation for 30 s, thereby activating the CRISPR-Cas12a system. 100% sensitivity was achieved for the O gene and 95.6% for the N gene, suggesting that photoactivation can be an efficient strategy for PoC pathogen detection.

### 5.2 Integration of Microfluidics

Microfluidic systems have allowed for leveraging the unique properties of fluid flow at micro- or nanoscale and to develop micrototal analysis systems (μTAS) and lab-on-a-chip (LOC) devices.^112^ LOC and μTAS have been essential to the generation of paper-based microfluidics tests,^113^ 3D tumor models,^114^ and POC pathogen diagnostics.^115^ Furthermore, microfluidic systems offer miniaturized reactions, lowering the cost of reagents and the required sample volumes. They allow for the integration of small heating units, multiplexed microchannels or microchambers, and detection platforms for fluorescent, colorimetric, plasmonic, and electrochemical probes, thereby enhancing the functionality of miniaturized automated devices.^116^ As a result, microfluidics have enabled the development of multifunctional, fully automated PoC platforms.^117^ For example, a paper-based microfluidic platform utilizing RT-LAMP-integrated Cas12a was fabricated to detect SARS-CoV-2 in wastewater samples.^118^ Three primer sets targeting the N, E, and surface protein (S) gene regions of SARS-CoV-2 were used for RT-LAMP amplification, and the LODs were recorded as 25, 310, and 10 copies mL^–1^, respectively. Results were obtained using lateral-flow assay and fluorescence, providing versatility and ease of result interpretation. The paper-based device consisted of five components: (i) a large hydrophilic disc surrounded by hydrophobic regions, (ii) a small hole for liquid transport, (iii) a glass fiber disc, (iv) a rectangle channel allocating the elution buffer into three paper discs, and (v) five paper discs (three for sample loading and two for the positive and negative controls). A poly(methyl methacrylate) (PMMA) plate was constructed for the LAMP and CRISPR reactions, microfluidic channels were fabricated using wax-printed filter paper, and a glass fiber disc was implemented to absorb RNA. The test could be completed under 2 h without any complicated equipment and proved to have 97.7% sensitivity and 82% semiquantitative accuracy. While the study was focused on SARS-CoV-2 detection in wastewater samples, similar strategies could be employed for the detection of pathogens in human clinical samples.

A streamlined and easy-to-use platform to detect *Vibrio parahemolyticus*, a pathogen causing raw or undercooked shrimp-associated acute gastroenteritis, was developed.^119^ Reversible valves were integrated on a chip to enable control over fluid flow, thereby allowing sample processing, LAMP amplification, and Cas 12a detection reactions to take place sequentially.^120^ Another platform utilized the centrifugal force to control fluid flow and direction to enable multiplex testing of 16 samples for *Pseudomonas aeruginosa* by integrating recombinase aided amplification (RAA) and Cas12a on a centrifugal assisted microfluidic chip.^121^ Taking multiplex testing one step further, multiple samples can be analyzed, and the nucleic acid quantification can be obtained digitally. Moreover, a parallel multistep digital analysis (PAMDA) SlipChip microfluidic platform was developed to quantify the SARS-CoV-2 genome in samples.^122^ Using simple slipping operations, LAMP and Cas12a reactions were conducted, and result interpretation can be completed using a smartphone. Hence, the integration of microfluidics can remarkably advance the functionalities of LAMP-integrated CRISPR platforms.

### 5.3 Development of Miniaturized Machines

Advances in nanotechnology, the development of microelectromechanical systems (MEMS) and nanoelectromechanical systems (NEMS), and the integration of light-, temperature-, enzyme-, and ion-responsive materials into conventional devices have motivated the generation of miniaturized machines.^123−125^ By manufacturing devices and tools in the micrometer to millimeter size range, micromachining techniques have enabled the fabrication of microscale devices with electrical and mechanical components, giving rise to MEMS and NEMS. Specifically, biomedical MEMS (BioMEMS) have been used for bioparticle manipulation,^126^ biological monitorization,^127^ and physical sensing.^128^ Moreover, BioMEMS have also been useful in the development of PoC diagnostic platforms, as they have effectively reduced sensor size and sample and reagent volumes and offered portability. For example, a micro-PCR device quantifying the human immunodeficiency virus (HIV) load in whole blood or plasma samples was developed.^129^ The device offered automated on-site testing with a rapid turnaround time for minimally trained users. In addition to the development of micro-PCR devices, the development of INAA devices has also led to remarkable outcomes. Unlike micro-PCR devices, INAA devices did not require thermal cycling features and appeared to be more suitable for PoC. A typical INAA device design would integrate sample preparation, amplification, and detection steps. For instance, an integrated glass microdevice was developed to enable the purification of nucleic acids, followed by their amplification using LAMP and finally fluorescent detection.^130^ Similarly, to detect IAV and IBV in less than 15 min, an INAA employing nicking endonuclease amplification reaction (NEAR) was developed.^131^ Another device offering rapid colorimetric detection of the IAV and methicillin-resistant *S. aureus* (MRSA) was controlled by a smartphone to provide ease of application.^132^ Evidently, several strategies have been employed to enhance the functionality of the INAA devices. Many of these strategies can be used to improve LAMP-integrated CRISPR assays and to increase access to rapid diagnostics in low-resource settings.

### 5.4 Machine Learning, Deep Learning, and Blockchain

The use of artificial intelligence (AI) in healthcare has remarkably advanced the collection and interpretation of data. Moreover, AI and computational biology have been translated into various applications in healthcare from cell counting to glioma classification.^133^ These applications were taken a step further to enable pattern recognition and interpretation of a huge magnitude of patient data using machine learning (ML).^134^ Beyond histopathology, ML has been used for omics,^135^ epidemiology,^136^ and the development of “intelligent” biosensors.^137^ In addition to reducing background noise and optimizing analyte signals, ML-integrated biosensors can offer single-molecule detection and improved accuracy. Similar to ML, the integration of deep learning (DL) have been used for improved data visualization such as holographic image reconstruction.^138^ DL has also been employed to provide a fast and efficient radiological imaging interpretation. Chest X-rays and computerized tomography (CT) scans have been used extensively for SARS-CoV-2 patients; however, conventional interpretation of these imaging modalities have not been sufficient for diagnosis.^139^ Classification-, detection-, and segmentation-based DL approaches have been employed to differentiate between SARS-CoV-2, pneumoniae, and healthy lung radiological images.^140−142^ ML/DL applications are not limited to data visualization and interpretation. Moving beyond individual-level diagnostics, PoC devices can benefit from integrated blockchain technology.^143^ In addition to electronic health records (EHS), blockchain-integrated sensors have been developed to ensure pharmaceutical and/or food safety throughout the supply chain.^144^ Furthermore, blockchain can be integrated into PoC devices as decentralized and interoperable systems that do not compromise patient privacy and data-security. Thus, blockchain-integrated PoC devices can be used to rapidly generate maps of outbreaks and inform public health measures.

## 6 Conclusion

Emerging infectious diseases continue to be a pressing global health challenge. Rapid and accurate identification of infected individuals is paramount to ensure that patients receive rapid diagnosis and that transmission between individuals is minimized. To achieve rapid and accurate pathogen detection globally, sensitive, specific, rapid, low-cost, and easy-to-use PoC platforms should be developed. While conventional methods such as cell culture, cytopathology, serological analysis, and NAATs offer benefits, they are not ideal for PoC applications, as they require either a long sample processing time or high-cost equipment. LAMP-integrated CRISPR-based pathogen detection has recently emerged as a promising method for PoC test development, providing versatility, high sensitivity, high specificity, simple result interpretation, and a rapid turnaround time. By integration of engineered Cas proteins and/or microfluidics, LAMP-integrated CRISPR detection can be optimized to reduce cost and maximize sensitivity, specificity, and convenience. Miniaturized devices can enhance the functionality of platforms at PoC settings. ML/DL integration can allow for improved data visualization and interpretation, and integration of blockchain can help guide public health efforts during outbreaks. LAMP-integrated CRISPR is a promising method for fully automated PoC pathogen detection applications in resource-limited PoC settings.
